# Microbial Siderophores for Sustainable Agriculture: Stress Tolerance, Biocontrol, Mechanism, and Omics Integration

**DOI:** 10.3390/ijms27219721

**Published:** 2026-10-29

**Authors:** Nahid Akhtar, Yogesh Ahlawat, Anamika Singh, Atif Khurshid Wani, Siva Prasad Panda, Arvind Kumar, Leena J. Patel, Piergiorgio Stevanato, Silvia Celletti

**Affiliations:** 1 Department of Microbiology, Graphic Era (Deemed to be University), Dehradun 248002, Uttarakhand, India; nahyd.akhtar24@gmail.com; 2 School of Allied and Health Sciences, CGC University, Mohali 140307, Punjab, India; assistantdean.ahs@cgcuniversity.in; 3 Department of Biotechnology, Vinayak Vidyapeeth College, Chaudhary Charan Singh University, Meerut 250110, Uttar Pradesh, India; asoni620@gmail.com; 4 Division of Veterinary Biochemistry, Faculty of Veterinary Sciences and Animal Husbandry, Sher-E-Kashmir University of Agricultural Sciences and Technology of Kashmir (SKUAST-K), Srinagar 190006, Jammu and Kashmir, India; atifkhurshid61200216@gmail.com; 5 Institute of Pharmaceutical Research, GLA University, Mathura 281406, Uttar Pradesh, India; sivaprasad.panda@gla.ac.in; 6 Department of Biotechnology, Noida Institute of Engineering and Technology, 19, Knowledge Park-II, Institutional Area, Greater Noida 201306, Uttar Pradesh, India; arvindumar.bio@niet.co.in; 7 Faculty of Science, Gokul Global University, Sidhpur 384151, Gujarat, India; hod.micro.gsc@gokuluniversity.ac.in; 8 Department of Agronomy, Food, Natural Resources, Animals and Environment, University of Padova, 35020 Legnaro, Italy; stevanato@unipd.it; 9 Department of Agricultural, Forest and Food Sciences (DISAFA), University of Turin, Largo Paolo Braccini 2, 10095 Grugliasco, Italy

**Keywords:** siderophores, biotic stress, abiotic stress, rhizosphere microbiome, sustainable agriculture, omics

## Abstract

Microbial siderophores play an important role in improving plant tolerance to abiotic and biotic stresses. Beyond iron acquisition, they support plant health by regulating oxidative stress, chelating toxic metals, and mediating beneficial plant–microbe interactions. This review highlights the role of siderophore-producing microbes in mitigating salinity, drought, alkaline stress, and metal toxicity, as well as in the improvement of nutrient solubilization and uptake. It also delineates environmental and physiological factors affecting siderophore production. The review additionally explores how the microbial interactions within soil consortia moderate siderophore biosynthesis and bio-control activity. Advances in multi-omics and genome editing approaches are discussed for identifying important genes and pathways associated with siderophore-mediated stress tolerance. The mechanisms behind siderophore-mediated stress tolerance are discussed, mainly their involvement in pathogen suppression through iron competition and activation of induced systemic resistance. Furthermore, it addresses the commercialization challenges and integration of siderophore applications into precision agriculture, aligning their agricultural and environmental benefits with the United Nations Sustainable Development Goals.

## 1. Introduction

Iron is vital for plant nutrition, growth, and health because it is essential for chlorophyll synthesis and photosynthesis. Lack of iron in plants can impair these processes, stunting plant development and growth. Therefore, iron deficiency can be a considerable challenge to agricultural productivity [1]. Microbes produce siderophores that bind to Fe^3+^ in iron-limited soils, thereby enhancing iron availability to plants [2]. Siderophores are produced by several bacteria, fungi, and plants [3]. The siderophores produced by plant fungal pathogens and bacteria aid in their virulence and in planta growth [4,5]. Siderophores are structurally diverse and are categorized into five main groups based on the type of moiety involved in iron coordination: carboxylates (e.g., staphyloferrin from *Staphylococcus* spp.), catecholates (e.g., enterobactin from enteric bacteria and *Streptomyces* spp.), hydroxamates (e.g., alcaligin from *Alcaligenes denitrificans*), mixed types (e.g., aerobactin from *Enterobacter* spp.), and phenolates (e.g., yersiniabactin from *Yersinia pestis*); see Table 1. The types of siderophores and their biosynthesis have been previously reviewed in detail [3]. Most documented siderophore producers are mesophilic bacteria that thrive at moderate temperatures, especially between 25–37 °C. However, numerous psychrotolerant and psychrophilic microorganisms have also been identified as siderophore producers [6].

Siderophores play a crucial role in a plethora of processes like competition among pathogens, signaling across different biological kingdoms, iron cycling in soils, and promotion of plant growth [7]. Siderophores are best known for their high affinity for iron, but they can also bind various other metals. This versatility assists siderophores to solubilize and mobilize heavy metals and metalloids in the soil, implying their potential application in the field of bioremediation [8]. Siderophore production and utilization can be influenced by environmental pH, as iron is readily available in soluble ferrous form (Fe^2+^) under acidic conditions but becomes poorly soluble ferric iron (Fe^3+^) in neutral to alkaline soils [9]. Other factors such as plant iron-acquisition strategy, siderophore structure, ligand exchange or reduction, and rhizosphere conditions can also affect siderophore production and utilization.

Siderophores are important for bacterial colonization, survival, and adaptation in plants. In bacteria like *Salmonella enterica*, siderophore production is necessary for the effective colonization, survival, and growth in association with plants [10]. The siderophore rhizobactin 1021 has been reported to be necessary for biofilm formation of *Sinorhizobium meliloti* on root and glass surfaces [11]. The siderophore desferrioxamine supported bacterial survival under nutrient-limited conditions in plant tissues and protected against reactive oxygen species [12]. A few siderophores, such as staphyloferrin B, are essential for colonizing the phyllosphere and solubilizing insoluble lanthanide oxides [13]. Siderophores act not only as competitive tools for iron acquisition but also enhance signals that promote beneficial microbes and strengthen plant defense responses [14].

**Figure 1:**
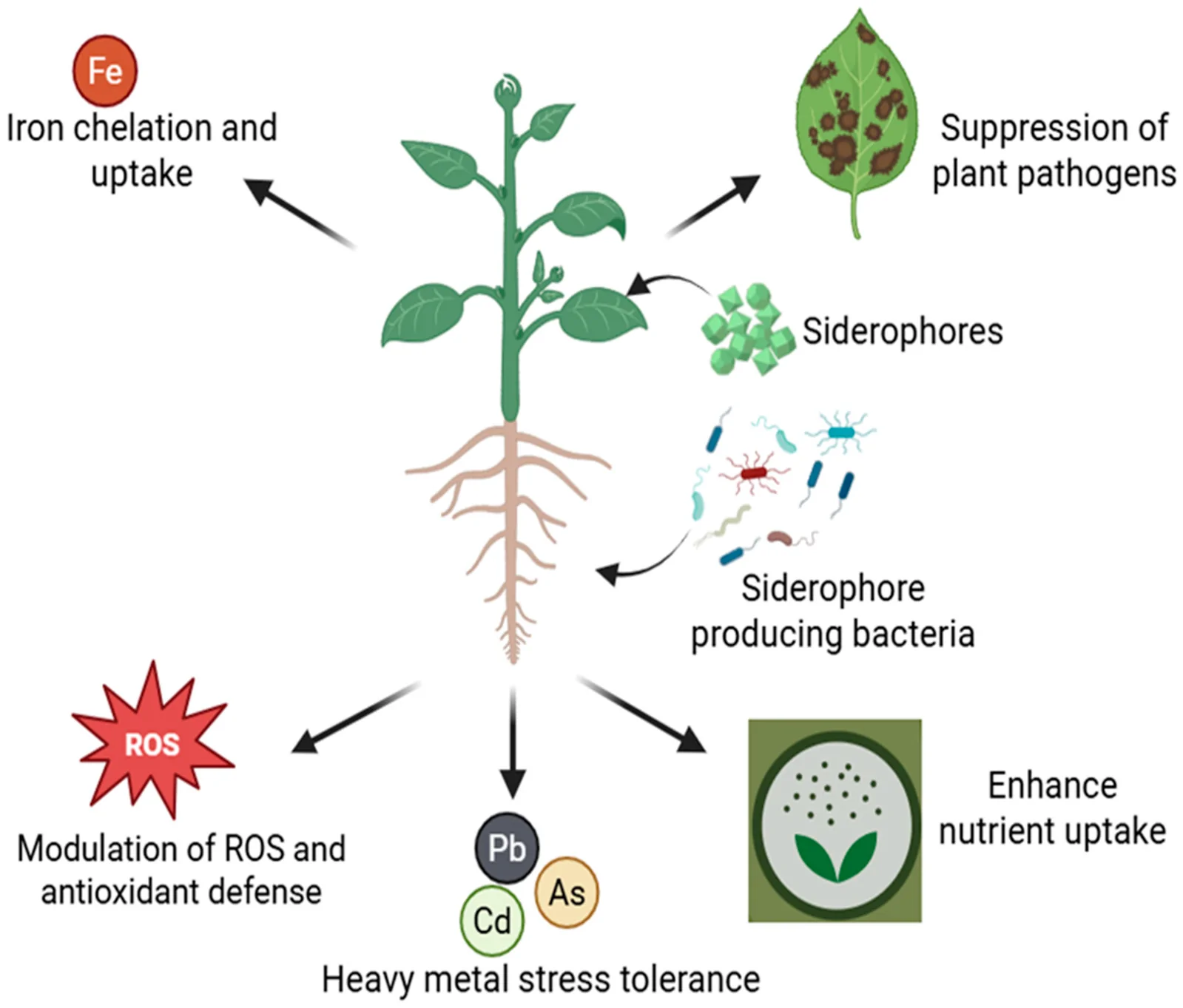
Roles of siderophores and siderophore-producing microbes in plant growth and stress tolerance.

The increasing global demand for food production, along with the adverse effects of climate change and soil degradation, requires the development of sustainable agricultural practices. Traditional farming methods are heavily reliant on excessive use of chemical fertilizers and pesticides, eventually contributing to environmental pollution and reduction in microbial diversity [15]. In this context, microbial siderophores offer a promising eco-friendly alternative to improve plant health, enhance soil fertility, and alleviate the effects of biotic and abiotic stresses (Figure 1). This review highlights the versatility of siderophores in plant–microbe–soil systems, identifying their contribution beyond iron acquisition to nutrient solubilization, bioremediation, stress signaling, and biocontrol of plant pathogens. The authors explore how siderophore-producing bacteria can improve plant resilience to salinity, drought, metal toxicity, and alkaline stress by modulating physiological and biochemical responses. The role of siderophores in disease suppression is examined. Numerous case studies are presented, covering the effects of specific siderophores (e.g., pyoverdine, desferrioxamine, bacillibactin) and microbial strains across a wide range of plant species and environmental conditions.

Siderophore production can contribute to diverse microbial interactions, with consequences that depend on the microorganism, host, and environmental context. Although siderophores produced by beneficial microorganisms may support plant growth, nutrient acquisition, and pathogen suppression, siderophore-mediated iron acquisition can also contribute to the survival and virulence of plant or human pathogens. Therefore, siderophore production alone should not be considered an indicator of agricultural suitability. The potential use of siderophore-producing microorganisms in agriculture requires strain-level evaluation of their plant-growth-promoting properties, pathogenicity, and biosafety, together with assessments of potential risks to plants, animals, humans, and the surrounding microbial community.

Previous reviews have examined microbial siderophores from several perspectives, including their biotechnological applications in agriculture, medicine, and bioremediation; their classification, biosynthesis, and role in plant growth promotion; their diversity, uptake pathways, and potential applications; and their involvement in the mobilization of heavy metals and the degradation of environmental contaminants [1,3,8,16,17]. While these reviews provide important foundations for understanding siderophore biology and its applications, the present review adopts a more integrated perspective on the regulation and agricultural functions of microbial siderophores. Specifically, it emphasizes (i) factors influencing siderophore biosynthesis under environmental fluctuations, (ii) microbial interactions and molecular cross-talk governing siderophore production and plant health, and (iii) insights from multi-omics and genome-editing approaches for understanding siderophore-mediated plant stress tolerance. By bringing these themes together, this review highlights emerging research directions, mechanistic uncertainties, and knowledge gaps relevant to the development of siderophore-based strategies for sustainable agriculture.

**Table 1:** Diversity of siderophores produced by different microorganisms.

Siderophore (Chemical Class)	Secreting Bacteria	Reference
Achromobactin (citrate-carboxylate siderophore)	*Pseudomonas syringae* B728a	[18]
Aerobactin (citrate-hydroxamate siderophore)	*Yersinia pseudotuberculosis*	[19]
Agrobactin (catechol)	*Rhizobium radiobacter*	[20]
Bacillibactin (catechol-type siderophore) and tribenglthin A	*Bacillus subtilis* NPROOT3	[21]
Bacillibactins E (nicotinic moiety) and F (benzoic acid moiety)	*Bacillus* sp.	[22]
Chrysobactin (catecholate siderophore)	*Dickeya chrysanthemi* EC16, *Erwinia chrysanthemi*	[23,24]
Chryseochelin A (citrate-based siderophore)	*Chryseobacterium* spp. CHR2, CHR5 and CHR6	[25]
Desferrioxamine (hydroxamate-type)	*Erwinia amylovora*	[26]
Amphibactins (tris-hydroxamates), crochelin A (mixed Fe-chelating groups), vibrioferrin (hydroxy carboxylate)	*Azotobacter chroococcum* NCIMB 8003	[27]
Fabrubactin A and B	*Agrobacterium fabrum* C58	[28]
Ferrioxamine B, D1, D2 and E	*Streptomyces ciscaucasicus* GS2	[29]
Frederiksenibactin (catechol-type siderophore)	*Yersinia frederiksenii* ATCC 33641	[30]
Gramibactin (N-nitrosohydroxylamine diazeniumdiolate moiety	*Paraburkholderia graminis*	[31]
Oosporein	*Chaetomium cupreum*	[32]
Pseudomonine	*Pseudomonas monteilii* MN759447	[33]
Pyochelin (hydroxyphenylthiazolinylthiazolidine type siderophore) and the rhamnolipid Rha-Rha-C15-C14	*Burkholderia seminalis* TC3.4.2R3	[34]
Pyoverdine (mixed type) and achromobactin	*Pseudomonas syringae* 1448a	[35]
Pyoverdine and pyochelin	*Pseudomonas aeruginosa* GRC1, *Pseudomonas putida*	[36,37,38]
Pseudobactin (hydroxamate type)	*Pseudomonas putida* ATCC 39167 and *Pseudomonas fluorescens* A225	[39]
Serobactin (lipopeptide siderophore)	*Herbaspirillum seropedicae*	[40]
Rhizobactin	*Sinorhizobium meliloti*	[11]
Triacetylfusigen, deshydroxylferritriacetylfusigen, desferritriacetylfusigen (members of fusarinine siderophore)	*Paenibacillus triticisoli* BJ-18	[41]
Triabactin and mupirochelin	*Pseudomonas* sp. NCIMB 10586	[42]
Variochelins A-E (lipohexapeptide siderophores containing hydroxamate and β-hydroxyaspartate functional groups	*Variovorax* sp. H002	[43]

Note: Some siderophore-producing microorganisms listed in this table include opportunistic or pathogenic species that may pose biosafety risks.

## 2. Literature Search and Selection Methods

The literature search was conducted to identify studies addressing the roles of microbial siderophores in sustainable agriculture, with particular emphasis on plant growth promotion, biocontrol, abiotic-stress tolerance, multi-omics approaches, and genome editing. Relevant literature was searched using freely available databases, namely PubMed and Google Scholar. The search covered publications from 2010 to May 2026 by applying a specific set of keywords. Selected earlier publications were also included when they were considered relevant to the scope of the review. These studies were identified by examining the reference lists of relevant articles, allowing important foundational research and earlier contributions to the understanding of microbial siderophores to be incorporated. The search was conducted using “microbial siderophores” and “siderophores” as the primary search terms, either independently or in combination with the following keywords: “plant growth promotion,” “biological control,” “drought stress,” “salinity stress,” “heavy metal stress,” “microbial interactions,” “microbial cross-talk,” “genomics,” “transcriptomics,” “multi-omics,” and “genome editing”. Studies were selected based on their relevance to the scope of the review, including primary research articles investigating siderophore production, biosynthetic pathways, plant–microbe interactions, pathogen suppression, abiotic-stress responses, and molecular or omics-based characterization. Relevant review articles were also considered to provide background information, identify developments in the field, and contextualize emerging research directions. Articles that were not in English were excluded. The literature was organized into thematic categories covering siderophore-mediated plant growth promotion and nutrient acquisition, biocontrol, abiotic-stress tolerance, microbial interactions, omics and genome-editing approaches, and agricultural translation. As a narrative review, the literature was synthesized qualitatively, with consideration given to the relevance of each study.

## 3. Role of Siderophores in Plant Growth Promotion and Nutrient Acquisition

Megapolibactins and plantaribactin, which are diazeniumdiolate-type siderophores, release nitric oxide in plant roots and can serve as biofertilizers since nitric oxide acts as a crucial plant hormone that regulates various plant functions such as defense, symbiosis, and growth [31]. Inoculating lentil seeds with the siderophore-producing bacterium *Bacillus subtilis* MT729775 increased nodule number, root length, shoot weight, and nitrogen and phosphorus uptake in *Lens culinaris* during pot culture experiments [44]. In another study, the bacteria *Bacillus methylotrophicus*, with siderophore-producing ability, enhanced shoot length, root length, tiller number, and dry weight of rice seedlings under both soil-less and pot conditions [45]. Similarly, in another study, the siderophore producer *Sphingomonas trueperi* NNA-14 increased root length, surface area, and fine roots of maize and increased nitrogen, calcium, sulfur, boron, copper, and zinc acquisition in maize shoots under greenhouse conditions [46]. Additionally, the cell-free supernatant rich in siderophores from the *Bacillus altitudinis* AS19 strain increased germination of maize and pepper seeds, as well as leaf and shoot development in *Gynura divaricate* [47]. These studies demonstrate the potential of siderophore-producing bacteria to improve plant growth and nutrient acquisition across different plant species. However, the results of whole-bacterium inoculation studies cannot be attributed exclusively to siderophores without additional experimental evidence, as these bacteria also produce phytohormones, ACC-deaminase, lipopeptides, antibiotics, and volatile organic compounds that help improve plant growth, increase stress tolerance, and prevent disease. Likewise, a cell-free supernatant enriched in siderophores may contain other extracellular metabolites that contribute to plant growth.

A more direct assessment of siderophore-mediated plant growth promotion comes from studies using siderophore preparations or purified compounds. The catecholate-type siderophore bacillibactin enhanced growth of *Malus hupehensis* seedlings and iron accumulation in calcareous soil and also influenced the rhizosphere microbial community [48]. Furthermore, a cell-free catecholate siderophore preparation from *Pantoea agglomerans* increased wheat plant biomass by 4.65% compared to iron-deficient control plants. In tomato, the preparation increased plant biomass by 51.5% and 1.8% compared with iron-deficient and iron-sufficient controls, respectively. Moreover, the catecholate siderophore boosted iron uptake in wheat, causing a 47.8% increase over iron-sufficient controls. Additionally, in tomato, chlorophyll content increased 4-fold with the catecholate siderophore compared to iron-deficient controls. Overall, the siderophore application outperformed bacterial cultures in several parameters in both tomato and wheat plants. Wheat, a Strategy II plant, showed greater response to the siderophore than Strategy I tomato, because of synergistic effects between microbial siderophores and wheat’s phytosiderophores. Furthermore, wheat exhibited higher iron translocation efficiency, exhibiting a greater tolerance to iron stress compared to tomato [2].

A recent study demonstrated that deferoxamine, a hydroxamate-type siderophore, promoted the growth of iron-deficient *Arabidopsis thaliana* plants by enhancing iron remobilization from roots to shoots and reducing oxidative stress in roots. These findings suggest that deferoxamine can contribute to plant growth under iron-limiting conditions by modulating internal iron homeostasis and mitigating oxidative damage [49]. One study reported that supplementing apple rootstocks with purified pyoverdine, a siderophore produced by *Pseudomonas* strain SP3, in hydroponic experiments under iron scarcity increased iron uptake and reduced chlorosis associated with iron deficiency [50]. Similarly, the supplementing *Zea mays* with a hydroxamate-type siderophore derived from *Synechococcus mundulus* promoted seedling growth under iron-deficient conditions and improved total carbohydrate, protein, carotenoid, and chlorophyll contents [51]. The use of siderophore preparations provides more direct evidence of their contribution to plant growth and nutrient acquisition than bacterial inoculation alone, particularly when the preparations are purified and appropriate iron-deficient and iron-sufficient controls are included. The reported increases in iron uptake, chlorophyll content, and biomass support a role for siderophores in alleviating iron deficiency. However, the magnitude of these responses may depend on plant species, iron status, siderophore type, and the availability of other nutrients.

Siderophores may also contribute to phosphorus acquisition by mobilizing phosphorus associated with poorly soluble mineral compounds. The arsenic-resistant bacteria, *Pseudomonas* sp. PG12, has been reported to solubilize phosphorus from phosphate rock and FePO_4_. Siderophores played an important role in phosphorus solubilization from these sources, as the bacteria had 10 times the solubilization potential of the mutant siderophore-producing bacterium *Pseudomonas fluorescens* Pf5 (PchA), which was impaired in siderophore production. The root and shoot biomass in PG12 treatment was 2.3- to 4-fold (0.04–0.07 g per plant) higher than the control (*Pseudomonas chlororaphis*) and PchA mutant. The increased tomato biomass was likely to be associated with enhanced phosphorus and iron uptake in the plant. The shoots and roots with PG12 treatment had 1.5–2.7-fold higher phosphorus concentration (7.46–11.6 mg g^−1^) than that in the control, and 1.3- to 1.7-fold higher than that of the mutant PchA. The Fe concentrations in tomato plants also increased 10-fold (10.8 mg/kg) in the shoots and 2-fold (270 mg/kg) in the roots with PG12 treatment compared to the PchA mutant [52]. The comparison between a siderophore-producing strain and a siderophore-biosynthesis-impaired mutant provides stronger mechanistic evidence than inoculation studies that lack such comparisons. The findings reported for *Pseudomonas* sp. PG12 support the involvement of siderophore production in phosphorus solubilization and improved phosphorus and iron acquisition. Further, genetic complementation or additional siderophore-specific experiments would further strengthen the causal interpretation.

The bacterial siderophores may also play a crucial role in improving plant nutrition during intercropping practices. Pyoverdine, a siderophore secreted by *Pseudomonas* spp., played a vital role in iron nutrition in intercropped maize and peanut [53]. The study reported that inoculating peppermint plants with the siderophore-producing *Pseudomonas putida* strain SJ04 resulted in a 60% increase in total essential oil content and a 7-fold increase in menthol content in the shoots compared to control plants [54].

The available evidence supports the potential of siderophores to improve plant growth and nutrient acquisition, particularly through their role in iron availability and, in some cases, phosphorus solubilization. Purified-siderophore treatments and comparisons involving siderophore-biosynthesis-impaired mutants provide stronger evidence for siderophore-specific effects than studies based solely on inoculation with siderophore-producing bacteria. Improvements in plant biomass, nutrient uptake, stress tolerance, or product quality following bacterial inoculation cannot be attributed solely to siderophores, as the inoculated bacteria may also produce other plant growth-promoting compounds. Further research involving siderophore-deficient mutants, genetic complementation, and purified-siderophore treatments is needed to clarify the specific contributions of siderophores to plant growth promotion.

## 4. Biocontrol Potential of Siderophore-Producing Microorganisms Against Plant Pathogens

Siderophores and siderophore-producing bacteria play a crucial role in suppressing plant pathogens (Table 2). However, the strength of the evidence varies among studies, depending on whether pathogen inhibition was demonstrated using purified siderophores, siderophore-enriched preparations, or whole bacterial cultures.

Several studies have demonstrated the direct inhibitory effects of siderophores or siderophore-containing preparations against plant pathogens under in vitro conditions. The 2-oxoisocaproate siderophore produced by *Proteus* sp. thwarted the Fusarium wilt disease caused by *Fusarium oxysporum* and promoted the growth of *Phaseolus mungo* in vitro [55]. Partially purified hydroxamate-type siderophores produced by *Pseudomonas aeruginosa* JAS-25 have been reported to limit the growth of the plant pathogens *Aspergillus niger* and *Fusarium udum* by inhibiting sporulation and degrading fungal mycelia in vitro [56]. The catechol-type siderophore (55.27 μg/ml) produced by *Pseudomonas syringae* BAF.1 inhibited the spore germination and growth of *F. oxysporum* under in vitro conditions [57]. Chryseochelin A obtained from *Chryseobacterium* spp. inhibited the phytopathogen *Ralstonia solanacearum* growth in a concentration-dependent manner by restricting access to iron in an in vitro experiment [25]. The carboxylate-type siderophore synthesized by *Pseudomonas mediterranea* inhibited the growth of *Phytophthora parasitica* var. nicotianae under low iron conditions in liquid Sucrose-L-Asparagine media [58]. Similarly, the pseudomonine siderophore-producing *Pseudomonas monteilii* strain MN759447 restricted the growth of *Talaromyces verruculosus, Talaromyces pinophilus, Fusarium oxysporum*, and *Aspergillus calidoustus* that increase plant mortality in *Dalbergia sissoo* [33]. The hydroxamate siderophore produced by *Burkholderia cepacia* XXVI inhibited *Colletotrichum gloeosporioides*, which causes anthracnose in mango [59].

**Figure 2:**
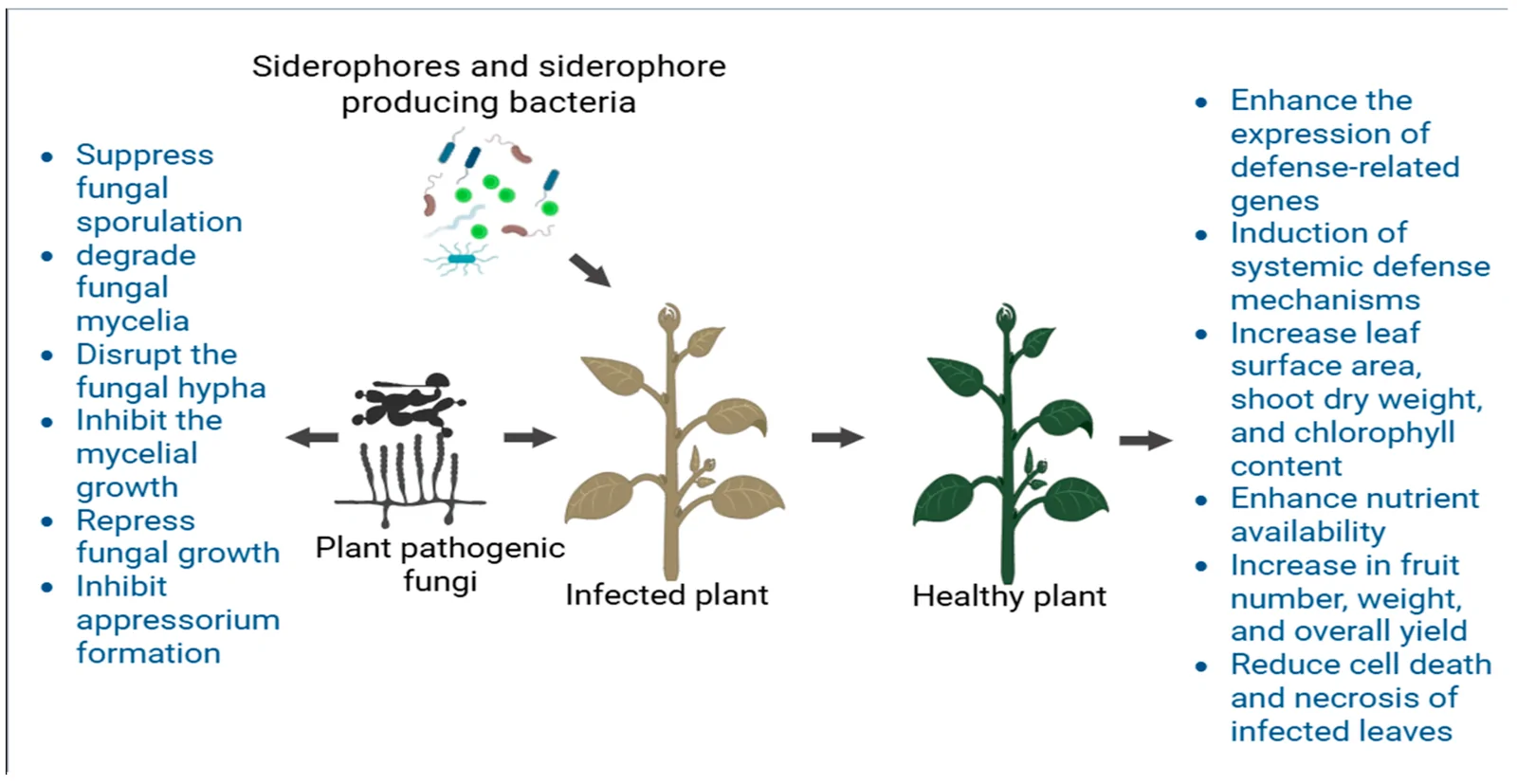
Effects of siderophores and siderophore-producing bacteria on fungal pathogens and their beneficial effects on plant health.

Evidence of siderophore-associated pathogen suppression has also been reported in plant-based assays. In a recent study, the use of a cell-free extract rich in siderophores such as ornibactin C8 and pyochelin, obtained from *Burkholderia contaminans* MSR2, ameliorated the effects of anthracnose caused by *Colletotrichum gloeosporioides* in avocado fruits in an in vivo antifungal assay [60]. The pyochelin-producing bacterium *Burkholderia pyrrocinia* BX1 effectively inhibited the growth and appressorium formation of *Colletotrichum fructicola*, the pathogen causing banana anthracnose. Moreover, the *B. pyrrocinia* BX1 filtrate repressed the expression of virulence genes in *C. fructicola* while increasing the expression of disease resistance genes in banana fruits [61]. Applying the bacillibactin siderophore, produced by *Bacillus subtilis*, to detached tomato and eggplant leaves infected with the plant pathogens *Fusarium equiseti* and *Alternaria porri* disrupted fungal hyphal structure and membrane integrity. Furthermore, treatment with the siderophore on infected leaves reduced cell death and necrosis associated with these fungal pathogens (Figure 2) [62]. These findings provide evidence of activity in postharvest or detached-leaf systems, although such assays do not by themselves establish efficacy under field conditions.

Some studies have further investigated siderophore-associated biocontrol in greenhouse, pot, or other in vivo experiments. The rhizobacteria *Bacillus velezensis*, as well as its catecholate-type siderophore bacillibactin, mitigated the harmful effects of *Meloidogyne incognita*, a root-knot nematode in banana plants. The in vitro experiments showed higher mortality of nematode juveniles under iron-limited conditions due to increased siderophore production. Furthermore, 500 μM of the purified bacillibactin was reported to be lethal to the nematode juveniles. Moreover, applying the siderophore (40 μmol/kg per 14 days) in greenhouse experiments reduced root gall formation, egg mass, and nematode population density and stimulated banana plant growth [56]. In another study, the siderophore produced by *Pseudomonas fluorescens* NK4 demonstrated potential as both a biocontrol agent and biofertilizer. The treatment of cucumber seedlings with a suspension of *P. fluorescens* NK4 and its siderophore improved survival rates against soil-borne pathogens. Furthermore, seedlings treated with *P. fluorescens* NK4 and its siderophore exhibited improved vegetative growth, as observed by increased leaf surface area, shoot dry weight, and chlorophyll content, largely due to increased nutrient and iron availability. Moreover, the treatments positively affected cucumber fruit production, leading to greater fruit number, weight, and overall yield compared to water-irrigated controls. Supplementing the treatments with ZnO nanoparticles further increased the secretion of fluorescent siderophores by *P. fluorescens*, leading to higher shoot dry weight, chlorophyll content, and fruit yield and quality [63]. The application of siderophore produced by *P. aeruginosa* JAS-25a led to an increase in seed germination, shoot length, root length, and dry weights in *Arachis hypogaea* (groundnut), *Cajanus cajan* (pigeon pea), and *Cicer arietinum* (chickpea) under pot study conditions [64]. Siderophore-producing strains *Alcaligenes faecalis* Bk1 and P1, along with *Bacillus amyloliquefaciens* Bk7, also inhibited the mycelial growth of *Fusarium graminearum*, *Botrytis cinerea*, *Rhizoctonia solani*, and *Magnaporthe oryzae*. The application of these bacterial strains reduced the incidence of rice blast and sheath blight in greenhouse experiments. Additionally, they enhanced the expression of key defense-related genes in rice, such as *OsWRKY45*, enriched mineral nutrient content in seedlings, and promoted plant growth [65].

Tomato plants pretreated with a siderophore-enriched, cell-free supernatant from *Acinetobacter baumannii* CBIO117 demonstrated a marked reduction in the severity and incidence of tomato vascular wilt disease caused by *F. oxysporum*. The treatment upregulated the expression of *ERF1* and *PR1* genes linked to ethylene and salicylic acid pathways. Furthermore, the study suggested that the disease-resistance effect is due to the induction of systemic defense mechanisms involving ethylene and salicylic acid, along with a priming effect via the jasmonic acid pathway, enhancing the plant’s resilience to phytopathogen attack [66]. The bacillibactin siderophore produced by *Bacillus velezensis* YL2021 effectively controlled rice blast disease caused by *Magnaporthe oryzae* and rice sheath blight caused by *Rhizoctonia solani* in pot experiments [67].

Inhibition by purified siderophores, as reported for bacillibactin and chryseochelin A, provides more direct evidence of siderophore activity than studies relying solely on bacterial inoculation [25,56]. The results obtained using cell-free extracts, culture filtrates, or siderophore-enriched supernatants cannot necessarily be attributed exclusively to siderophores unless their contribution has been experimentally isolated. Future studies using siderophore-deficient mutants, genetic complementation, and purified compounds would help establish causal mechanisms. Furthermore, although in vitro inhibition demonstrates antimicrobial potential, detached-leaf, postharvest, greenhouse, and pot studies provide different levels of evidence for practical application; field trials are needed to determine the consistency and effectiveness of these approaches under agricultural conditions. Despite their promising biocontrol potential, the agricultural application of siderophore-producing microorganisms must be evaluated carefully, particularly when the strains belong to species associated with human opportunistic infections, such as *Pseudomonas aeruginosa*, *Acinetobacter baumannii*, and certain *Burkholderia* species. Their potential use as agricultural inoculants should be preceded by rigorous strain-level biosafety assessment, including evaluation of pathogenicity, virulence-associated traits, antimicrobial resistance, and potential risks to humans, animals, and the environment.

**Table 2:** Effects of siderophore-producing microorganisms on plant pathogen suppression.

Pathogen/Disease	Plant	Microbial Strain	Evidence for Siderophore Involvement	Experimental System	Outcome	Reference
*Acidovorax citrulli*/Bacterial fruit blotch	*Cucumis sativus*	*Paenibacillus tianmuensis* YM002	Indirect **	In vitro	Decreased development of necrotic symptoms, inhibited pathogen growth and reduced virulence, enhanced plant growth	[68]
*Alternaria alternata*/Early blight	*Solanum lycopersicum*	*Streptomyces* strain AzR-051	Indirect	In vitro	Enhanced seedling germination, seed and root length, inhibited *A. alternata* growth	[69]
*Botrytis cinerea*/Gray mold	*Solanum lycopersicum*	*Bacillus velezensis* FX-6	Indirect	In vitro and in detached leaves	Increased seedling growth and seed germination, restricted *B. cinerea* invasion on tomato leaves, inhibited growth of 7 fungal phytopathogens in vitro	[70]
*Botrytis cinerea*	*Solanum lycopersicum*	*Pseudomonas putida* Z13	Indirect	In vitro, in planta	Upregulatation of the plant defense-related genes *PIN2* and *PR1*, inhibited the pathogen growth in vitro, reduced disease severity in plants	[71]
*Cephalosporium maydis*/Late wilt	*Zea mays*	*Pseudomonas koreensis* and *Bacillus subtilis*	Indirect	In vitro, in pot experiment, and in artificially infested field conditions	Improved plant growth, inhibited mycelial growth in vitro, provided resistance to late wilt, reduced damping off in pot experiments, reduced disease incidence and improved yield under field conditions	[72]
*Colletotrichum siamense*/Anthracnose	*Malus domestica* cv. Fuji	*Bacillus subtilis* GYUN-2311	Indirect	In vitro, and post-harvest apple fruits	Inhibited germination of *C. siamense* spores and appressorium formation in vitro, inhibited apple anthracnose in vivo	[73]
*Fusarium oxysporum/*Fusarium crown and root rot (FCRR)	*Solanum lycopersicum*	*Paenibacillus polymyxa* SC09-21	Indirect	In vitro, in vivo	Inhibited mycelial growth in vitro, suppressed FCRR in plants under greenhouse conditions	[74]
*Fusarium solani*/Fusarium wilt	*Cnidium officinale*	*Leclercia adecarboxylata* PT1	Indirect	In vitro, in planta	Inhibited fungal growth in vitro, Reduced shoot mortality rate and increased fresh and dry weights of shoots in fungi infected plants	[75]
*Fusarium temperatum/*Root rot	*Zea mays*	*Burkholderia cenocepacia* KNU17BI2	Indirect	In vitro, in vivo	Inhibited *F. temperatum* mycelial growth in vitro, suppressed root rot and improved plant growth in greenhouse experiments	[76]
*Macrophomina phaseolina/*Charcoal rot	*Glycine max*	*Bacillus subtilis*	Indirect	In vitro, in vivo under greenhouse conditions	Inhibited mycelial growth in vitro, reduced disease incidence in infected plants, improved growth of plants under greenhouse conditions	[77]
*Rhizoctonia solani and Phytophthora infestans/*black scurf and stem canker and late blight	*Solanum tuberosum*	*Bacillus subtilis* EG21	Indirect	In vitro, on detached leaves	Damaged *R. solani* mycelia in vitro, protected harvested leaves from *P. infestans* during leaf disk and detached leaf assays	[78]
*Rhizoctonia solani/*Root rot disease	*Solanum lycopersicum*	*Pseudomonas fluorescens* MPF47	Indirect	Plants in greenhouse and field experiments	Reduced root rot under glasshouse conditions, more effective in controlling the disease than carbendazim fungicide under field and greenhouse conditions, increased seedling growth and yield under greenhouse condition	[79]
*Ralstonia solanacearum/*Bacterial wilt	*Capsicum annuum*	*Pseudomonas fluorescens* PDS1 and *Bacillus subtilis* KA9	Indirect	In vitro, plants grown in glasshouse	Inhibited pathogen growth in vitro, reduced bacterial wilt incidence in plants under glasshouse conditions, provided resistance by increasing activity of peroxidase, polyphenol oxidase, superoxide dismutase and phenylalanine ammonium lyase in plants under glasshouse condition	[80]
*Sclerotium rolfsii/*collar rot	*Cicer arietinum*	*Pseudomonas isolates* P66, P141 and P200	Indirect	In vitro, in field trials	Inhibited fungal growth in vitro, reduced collar rot incidence and increased plant growth and yield in field trials	[81]
*Sclerotium rolfsii/*Pepper southern blight	*Capsicum annuum*	*Streptomyces griseoaurantiacus* XQ-29	Indirect	In vitro, potted seedling in greenhouse	Inhibited mycelial growth, sclerotia production and germination in vitro, reduced the pepper southern blight incidence in seedlings, activated cellular defense responses in plants pretreated with *S. griseoaurantiacus* XQ-29	[82]
*Xanthomonas oryzae/*Bacterial leaf blight	*Oryza sativa*	*Bacillus albus* and its siderophore extract	Direct *	In vitro and in vivo	*Bacillus albus* and its siderophore extract inhibited *X. oryzae* growth during both in vitro and in vivo conditions, increased length and weight of roots and shoots in treated plants compared to control plant infected with *X. oryzae* under in vivo evaluation	[83]

* Direct evidence refers to studies that specifically investigate siderophore-mediated pathogen suppression using purified siderophores or siderophore extracts. ** Indirect evidence refers to studies reporting pathogen suppression by siderophore-producing microorganisms without experimentally establishing the specific contribution of siderophores to the observed effects.

## 5. Siderophore-Mediated Abiotic Stress Tolerance

### 5.1. Siderophore-Mediated Alleviation of Heavy Metal Stress in Plants

Siderophores improve iron availability while also chelating heavy metals, thus reducing heavy metal toxicity [84]. Some siderophores show an affinity for divalent and trivalent cations such as Pb^2+^, Ni^2+^, Zn^2+^, Cu^2+^, Cd^2+^, and Al^3+^ (Table 3). These heavy metal–siderophore complexes are less toxic to cells compared to free metals because they are either less available or inaccessible to the producing microorganisms and surrounding organisms [85]. Siderophores increase auxin synthesis by chelating metal ions such as Ni^2+^, Cu^2+^, Cd^2+^, and Al^3+^, hence stimulating auxin production even in the presence of these metals [86]. The effects of siderophores on plant metal stress tolerance depend on the metal involved and the resulting changes in its availability, uptake, and distribution within the plant. Importantly, reduced metal accumulation and enhanced metal uptake represent different outcomes: the former may help protect crops from toxic metal accumulation, whereas the latter may be desirable for phytoremediation of contaminated environments.

Several studies have demonstrated improved plant growth and physiological performance under heavy metal stress following treatment with siderophore-producing microorganisms. Siderophores produced by *Streptomyces acidiscabies* E13 supplied iron to cowpea plants, even in the presence of metals like Al, Cu, Mn, and Ni that inhibit iron acquisition. The supplementation of siderophore improved chlorophyll content and prevented lipid peroxidation in leaves. Furthermore, the siderophore protected microbial auxins from degradation by reducing free radical formation, thereby increasing auxin-mediated plant growth and aiding increased metal uptake [87]. In another study, culture filtrates of nickel-resistant *Streptomyces acidiscabies* E13, containing hydroxamate siderophores, increased cowpea height and biomass under nickel stress, regardless of the plants’ iron status. The siderophores bound to iron and nickel, promoting cowpea growth by supplying iron but decreasing nickel uptake. This dual function supports iron availability for plant use while protecting against nickel toxicity [88].

Similarly, *Medicago sativa* seedlings inoculated with siderophore-producing bacteria *Pseudomonas japonica* isolates BEP17-Dm and BEP18-Dm, as well as *Bacillus subtilis* BRU16, isolated from the rhizosphere of heavy metal-tolerant plants, demonstrated higher survival rates under in vitro conditions in the presence of aluminum, cadmium, and lead [85]. The siderophore-producing bacteria *Pseudomonas fluorescens* has been shown to improve chickpea growth under cadmium stress, increase germination of chickpea under cadmium stress by 10%, the root and shoot dry biomass by 52% and 37%, and seed yield by 26% compared to plants exposed to cadmium stress without *P. fluorescens* treatment [89]. The treatment of maize seeds with the siderophore-producing *Pseudochrobactrum asaccharolyticum* isolated from arsenic-contaminated soil improved photosynthetic pigment levels, including chlorophyll and carotenoids, and increased internal CO_2_ concentration, stomatal conductance, and photosynthetic rate in maize plants under arsenic stress [90]. These studies demonstrate the potential of siderophore-producing microorganisms to improve plant performance under metal stress; however, improved growth or survival alone does not establish that toxic metal accumulation in plant tissues has been reduced.

In alfalfa, inoculation with siderophore-producing microbes like *S. meliloti* and *P. fluorescens* increased growth rate and reduced cadmium accumulation in shoots. Furthermore, root colonization by these bacteria also resulted in higher root dry weights. Additionally, co-inoculation with the endophytic fungus *Piriformospora indica* and these siderophore-producing bacteria further enhanced alfalfa performance under cadmium stress by improving uptake of growth-limiting nutrients, which led to an increase in overall plant biomass. The highest shoot dry weights were observed in plants treated with *P. fluorescens* in combination with *P. indica* under cadmium stress. *P. indica* and the siderophore-producing bacteria alleviated cadmium-induced oxidative stress by increasing the antioxidant enzymes’ activity (ascorbate peroxidase, guaiacol peroxidase, and catalase) [91]. The reduction in shoot cadmium accumulation is relevant to limiting metal transfer to aboveground plant tissues. However, the implications for food safety depend on the crop and the tissues intended for consumption.

In contrast to strategies intended to limit toxic metal accumulation in crops, siderophore-mediated metal mobilization may be useful for phytoremediation when the objective is to increase metal availability and plant uptake. Endophytes with high siderophore production can also help host plants in arsenic phytoremediation. Inoculation with *Enterobacter* sp. YG-14 increased arsenic bioaccumulation in *Robinia pseudoacacia*, mainly in the roots. Here, *Enterobacter* sp. YG-14 assisted the mobilization of iron-bound arsenic, transforming it into bioavailable arsenic through the siderophore enterobactin. Additionally, root colonization by this enterobactin-producing strain in *R. pseudoacacia* boosted antioxidant enzyme activity and increased glutathione (GSH) levels, implying the YG-14 strain’s capacity to ameliorate oxidative stress through root colonization [92]. Although increased arsenic accumulation may be beneficial for remediation, mobilization can also increase the risk of arsenic transfer into other environmental compartments or plant tissues.

Similarly, application of a consortium comprising siderophore-producing *Bacillus aerophilus* TR15c and ACC deaminase-producing *Pseudomonas* sp. TR15a to *Helianthus annuus* in pot experiments containing 100 mg Cu kg^−1^ increased copper uptake, seed germination, and biomass production [93]. This finding suggests potential for combining improved plant growth with enhanced copper uptake in contaminated environments. However, the contribution of siderophores must be distinguished from that of ACC deaminase and other microbial traits when interpreting the effects of the consortium. In another study, *Pteris vittata* grown in a solution containing catecholate-type siderophores produced by the arsenic-resistant *Pseudomonas* strain PG12, along with FeAsO_4_ mineral, showed increased arsenic and iron uptake and enhanced the plants’ growth [94]. These findings indicate that siderophore-mediated mobilization may support metal uptake by plants. Still, its effectiveness for phytoextraction depends on whether harvesting can remove the accumulated metal and whether the mobilization process creates additional environmental or food-safety risks.

The effects of siderophore-producing bacteria on metal uptake may also involve the acquisition of both essential nutrients and potentially toxic metals. The cobalt-tolerant, siderophore-producing bacteria *Bacillus albus* and *Bacillus subtilis* enhanced chlorophyll content, root and shoot length, and fresh and dry biomass in black gram plants under 2000 ppm cobalt stress. Moreover, the bacteria promoted nitrogen and phosphorus uptake by plant roots and also improved cobalt and iron accumulation in the roots compared to untreated controls. Cobalt’s competition with iron often reduces chlorophyll levels, leading to iron deficiency in plants. However, the siderophore production by the cobalt-tolerant bacteria likely improved iron availability and cobalt mobilization into plant roots, assisting in concurrent iron and cobalt uptake. The cobalt tolerance mechanisms of these bacteria, likely involving cytosolic sequestration or enzymatic cation management, helped mitigate cobalt stress while enriching the plants with essential nutrients nitrogen and phosphorus, and micronutrients cobalt and iron [95].

Overall, siderophores can alleviate heavy metal stress through several processes, including iron acquisition, metal complexation, changes in metal availability, and interactions with plant physiological and antioxidant responses. However, when these effects are observed following inoculation with siderophore-producing microorganisms, they cannot necessarily be attributed solely to siderophores, as other microbial traits may also contribute to the observed responses. Further studies using siderophore-deficient mutants, genetic complementation, and purified-siderophore treatments are needed to establish the specific contribution of siderophores to heavy metal stress tolerance. Nevertheless, their effects on metal accumulation are not uniform. Strategies that reduce metal uptake or limit its transfer to edible tissues may be appropriate for crop production in contaminated soil. In contrast, approaches that increase metal mobilization and plant uptake may be more suitable for phytoremediation. These objectives should be evaluated separately because enhanced metal availability, particularly for arsenic and cadmium, can increase food-safety risks if toxic metals accumulate in edible plant parts. The mechanisms of siderophore-mediated stress tolerance, including that of heavy metal stress, have been discussed in Section 6.2.

**Table 3:** Effect of siderophore-producing bacteria on plants grown under heavy metal stress.

Heavy Metal	Siderophore Producing Bacteria	Plant Species	Initial Heavy Metal Concentration and Growing Condition	Effect	Metal Distribution	Reference
Arsenic	*Acinetobacter lwoffii* RJB-2	*Vigna radiata* (mung bean)	Pots containing soil amended with 22.5 mg/kg sodium arsenate	Increased plant growth, carotenoid and chlorophyll content, and abated arsenic uptake in mung bean	In inoculated plants, arsenic content was reported as 0 μg/g in roots, stems, and leaves. In plants not treated with bacterial inoculum after 7 days, As contents were 501, 15.2, and 11.2 μg/g in roots, stems, and leaves, respectively	[96]
Arsenic	*Bacillus aryabhattai* AS6	*Oryza sativa* (rice)	Pots filled with soil containing 2.5 mM As(V)	Enhanced activity of germination related enzymes protease and amylase, increased seedling growth, increased superoxide dismutase and catalase activity in rice seedlings	Not available	[97]
Arsenic	*Bradyrhizobium japonicum* CB1809	*Glycine max* (soybean)	Nutrient solution containing 5 μM arsenic	Increased biomass of soybean roots	Not available	[98]
Cadmium	*Enterobacter* sp. Zm-123	*Brassica napus* (rapeseed)	0, 0.5, 1, 3, and 5 mg/L in hydroponic nutrient solution	Reduced Cd content and electrolyte leakage, increased peroxidase and catalase activity in rape seedlings	Cadmium content ranged between 0.25–0.85 mg/g, and 0.16–0.75 mg/g in the non-inoculated and inoculated seedlings respectively	[99]
Cadmium	*Pseudomonas rhodesiae*	*Arabidopsis thaliana*	MS agar media supplemented with 2.5 mg/L of CdCl_2_	Increased total length and fresh weight of hypocotyl, increased lateral root density and number in A. thaliana seedlings	Not available	[100]
Cadmium	*Streptomyces phaeogriseichromatogenes isolate* COS4	*Helianthus annuus (sunflower)*	HW agar media supplemented with 10 mg/L cadmium	Increased root to tip length and root length, total fresh and dry biomass of sunflower seedlings	Not available	[101]
Cadmium	*Burkholderia contaminans* JLS17	*Pinus elliottii* (slash pine)	3, 6 and 9 mg/kg cadmium in potting soil	Enriched the concentration of acid-soluble cadmium and decreased the concentration of reducible, oxidizable, and residual cadmium in cadmium contaminated soil, enhanced the growth of both the shoots and roots of *P. elliottii* under cadmium stress	Not available	[102]
Cadmium	*Bacillus strains* WL1210 and CD303	*Lolium perenne* (ryegrass)	20, 40, 60 and 80 mg/L cadmium mixed in potting soil	Promoted root length and plant height, and maintained normal photosynthesis activity of ryegrass	Not available	[103]
Chromium	*Bacillus subtilis* S2VWR5	*Cicer arietinum* (chickpea)	Soil in each pot contained 2 g Cr	Improved root and shoot length, biomass of chickpea	Approximately 0.4 and 0.6 µg/ml Cr accumulated in shoot and root of control plants respectively. In bacteria treated plant 2 µg/ml Cr accumulated in root, and in shoot approximately 0.45 µg/ml chromium accumulated	[104]
Chromium	*Pseudomonas* sp. NT27	*Medicago sativa* (alfalfa)	10 mg of 70 mg/kg of Cr(VI) per kilogram of soil in plastic pots	Enhanced root and shoot biomass, increased chlorophyll content, reduced proline and malondialdehyde level in *Medicago sativa*	Cr uptake (μg/g dry weight): Roots: not determined in the control, 3.12 in Cr(VI)-treated plants, and 4.65 in Cr(VI) + *Pseudomonas* sp. NT27-treated plants. Shoots: not determined in the control, 1.49 in Cr(VI)-treated plants, and 1.58 in Cr(VI) + *Pseudomonas* sp. NT27-treated plants after 45 days	[105]
Chromium	*Bacillus subtilis PAW3*	*Vigna unguiculata* (cowpea)	Pots containing soil with 70 mg/kg of Cr(VI)	Promoted growth and photosynthesis of cowpea plants	At an initial soil Cr(VI) concentration of 70 mg/kg, the uninoculated treatment retained 58 mg/kg of Cr(VI) in the soil, whereas the *B. subtilis* PAW3-inoculated treatment retained 62 mg/kg of Cr(III) in the soil suggesting reduction of Cr(VI) to Cr(III) form after 30 days of cowpea growth	[106]
Copper	*Stenotrophomonas maltophilia* RBTS7	*Zea mays* (maize)	2 g CuSO_4_ in 1 kg of soil in pots under greenhouse condition	Reduced copper induced toxicity, increased growth and increased copper uptake in stem and roots of maize	Control plants: approximately 58 mg/kg in roots and 25 mg/kg in shoots. Bacteria-inoculated plants: approximately 100 mg/kg in roots and 55 mg/kg in shoots	[107]
Lead	*Rhodococcus qingshengii* LMR356	*Sulla spinosissima*	Pots filled with soil collected from heavily lead-contaminated site	Enhanced biomass, promoted activity of antioxidant enzymes, increased carotenoid and chlorophyll content and stress tolerance	Not available	[108]
Lead	*Pseudomonas* sp. strain PG-12	*Pteris vittata*	Sterile potting medium spiked with 1 mM PbCl_2_	Enhanced growth, increased phosphorus uptake, and decreased lead accumulation in *Pteris vittata* tissues	More lead accumulated in the roots (128–262 mg/kg) than in the shoots (10–29 mg/kg)	[109]
Nickel	*Pseudomonas putida*	*Eruca sativa*	Pot containing sandy soil mixed with either 50 µg/g, 250 µg/g or 500 µg/g NiCl_2_	Increased root and shoot length, biomass, chlorophyll levels of *Eruca sativa*	Not available	[110]
Nickel	*Bacillus megaterium* AFI1	*Triticum aestivum* (wheat)	Knop solution containing 100 µM/L of nickel cations	Ameliorated nickel induced oxidative stress, increased growth and content of photosynthetic pigments in wheat plants	Bacterial inoculation reduced nickel content in the roots and shoots wheat cultivars	[111]

### 5.2. Potential Role of Siderophore-Producing Bacteria in Drought Tolerance

Drought tolerance in plants involves several physiological responses, including the maintenance of high plant water potential, osmolyte accumulation, activation of drought-induced proteins, changes in ion exchange, and antioxidant defense [112]. Siderophore-producing bacteria have been associated with improved plant growth under water-deficit conditions; however, the extent to which these effects can be attributed specifically to siderophores remains uncertain.

Several studies have reported improved plant growth following inoculation with siderophore-producing bacteria. The siderophore-producing bacteria *Paraburkholderia phytofirmans* LP-R1r, *Caballeronia udeis* LP-R2r, and *Caballeronia sordidicola* HP-S1r enhanced the length and biomass of their natural host, lodgepole pine, as well as a foreign host, hybrid white spruce, under xeric-cold conditions in greenhouse experiments [113]. Different siderophore-producing drought-tolerant *P. aeruginosa* strains increased drought tolerance and improved growth in *Vigna radiata* under drought conditions [114]. These findings indicate the potential of bacterial inoculation to improve plant performance under water-deficit conditions, although the reported growth responses alone do not establish the specific contribution of siderophores.

Evidence linking siderophore production to drought tolerance is also reported in studies examining seed germination and plant physiological responses. Colonization of maize and wheat seeds with the psychrotrophic bacterium *Pseudomonas protegens* LPH60 promoted seed germination and increased shoot and root length under PEG-6000-induced drought stress without causing phytotoxicity. The improvement in germination may be attributed to siderophore synthesis. The genome of *P. protegens* LPH60 includes genes encoding enzymes such as pyoverdine-tailoring dipeptidase-like protein, SidA/IucD/PvdA family monooxygenase, PvdJ/PvdD/PvdP-like protein, and isochorismate synthase, which are associated with siderophore biosynthesis [115]. However, the presence of siderophore biosynthesis-associated genes and the observed plant growth responses do not, by themselves, demonstrate that siderophores directly mediate drought tolerance.

Beyond growth and germination, some studies have reported changes in plant water status and antioxidant responses following inoculation with siderophore-producing bacteria. Wheat plants demonstrated improved resilience to water stress when treated with siderophore-producing bacteria *Bacillus safensis* and *Ochrobactrum pseudogregnonense*, as shown by delayed wilting, higher relative water content in treated stressed plants than in untreated ones, and higher antioxidant activity [116]. Another study showed that priming wheat seeds with siderophore-producing *Serratia marcescens* RRN II2 and *Pseudomonas* sp. RRC I5 bacteria improved water retention, increased osmolyte accumulation, and also enhanced chlorophyll and carotenoid levels in plants under drought stress [117].

Overall, these studies associate siderophore-producing bacterial inoculation with improved growth, water retention, osmolyte accumulation, and antioxidant activity under drought stress. Nevertheless, the evidence presented is predominantly based on inoculation studies, and the reported physiological responses cannot necessarily be attributed directly to siderophores because the bacteria may possess additional plant-growth-promoting traits. Currently, there is a lack of studies that directly link siderophores to drought tolerance in plants. Thus, the specific contribution of siderophores to drought tolerance remains to be established more conclusively.

### 5.3. Potential Role of Siderophore-Producing Bacteria in Salinity Stress Tolerance

Salinity stress affects plant growth through osmotic stress, ion toxicity, oxidative damage, and by affecting biochemical processes [118]. Researchers have investigated siderophore-producing bacteria as potential tools to mitigate these effects, with reported benefits including improved growth, changes in the potassium-to-sodium ratio, osmolyte accumulation, and enhanced antioxidant activity.

Several studies have reported improved plant growth following inoculation with siderophore-producing bacteria. The inoculation of barley and wheat seeds with siderophore-producing, salt stress-tolerant bacteria increased plant height and grain yield under high salt stress (8 dS/m NaCl) compared with the control plant [119]. Similarly, another study found that inoculating cucumber seedlings with salt-tolerant *B. subtilis* C8 improved leaf area, plant height, and dry and fresh weight under salinity stress (4, 8, and 16 dS/m NaCl) compared with the control plant [120]. Inoculation with the siderophore-producing *Pseudomonas protegens* CP17 caused improvement in biomass, growth, and germination of wheat seedlings under various NaCl concentrations (0 mM, 100 mM, and 150 mM) [121]. Similarly, 2 siderophore-producing *B. velezensis* strains (CSS_12 and PB_8) improved lettuce growth, increased photosynthetic pigment content, and reduced damage caused by drought and salt stress [122]. These studies demonstrate the potential of siderophore-producing bacterial inoculation to improve plant performance under salinity stress but do not establish the involvement of siderophores in salt tolerance.

Some studies have also reported changes in ion homeostasis and antioxidant responses in plants inoculated with siderophore-producing bacteria. A study indicated that inoculation with siderophore-producing *P. fluorescens* CECT 378(T) bacteria partially ameliorated salt stress (100 mM NaCl) in sunflower plants, increasing the K^+^/Na^+^ ratio in the shoots and improving plant resilience [123]. Treating rice plants with the siderophore-producing *Streptomyces griseoincarnatus* RB7AG increased proline and antioxidant enzyme accumulation (catalase, peroxidase, and superoxide dismutase), as well as root and shoot length, under salt stress [124]. The combined treatment of salt-tolerant, siderophore-producing *Enterococcus faecium* and *Pantoea dispersa* improved biomass, plant height, pod and seed count, and seed and pod weight in mungbean grown under saline stress (5 and 10 dS/m NaCl). The treatment also decreased membrane damage in leaves and increased levels of ascorbic acid and glutathione [125].

Further evidence of physiological changes under salinity stress comes from studies reporting increased osmoprotectant levels and antioxidant activity. Inoculation with the siderophore-producing *Bacillus anthracis* PM21 enhanced plant growth metrics, boosted antioxidant enzyme activity, increased osmoprotectant levels, and increased chlorophyll content under salt stress conditions (180, 240 and 300 mM NaCl) [126]. Although these findings suggest that inoculating with siderophore-producing bacteria can improve ion balance, osmolyte accumulation, and antioxidant responses under salinity stress, they do not necessarily establish that siderophores directly cause these effects. The observed benefits may also result from other plant growth-promoting traits of the inoculated bacteria. Future studies using siderophore-deficient mutants, genetic complementation, and purified-siderophore treatments, alongside appropriate controls, are needed to establish the causal role of siderophores in improving plant tolerance to salinity stress.

### 5.4. Potential Role of Siderophore-Producing Bacteria in Alkaline Stress Tolerance

Iron deficiency is a common challenge for terrestrial plants in alkaline soils because of iron’s low solubility under such conditions [127]. In response, salt-tolerant bacteria produce high-affinity siderophores that form stable, soluble complexes at alkaline pH. These Fe^3+^–siderophore complexes can be utilized by microbes and plants, which will improve iron availability in the rhizosphere [128]. Salt-tolerant, siderophore-producing *Enterobacter asburiae* A103 increased alfalfa growth, along with improvements in shoot and root dry weights and lengths under alkali stress (100 mM NaHCO_3_) [129]. Similarly, inoculation with *Pantoea eucalypti* M91, a plant growth-promoting bacterium producing pyoverdine- and pyochelin-like siderophores in alkaline environments, assisted in Fe^2+^ transport to the shoots, upregulated Fe uptake-related genes such as iron-regulated transporter 1 (*IRT1*) and ferric reductase oxidase 1 (*FRO1*), and acidified the hydroponic medium of *Lotus japonicus* cultures [130]. *Peribacillus frigoritolerans* TRM58009, a salt-tolerant bacteria isolated from saline meadow soils of the Pamir Plateau, has demonstrated potential in improving the tolerance of maize plants to alkaline and saline conditions. The bacteria produce hydroxamate-type siderophores that promote iron uptake in maize under saline-alkaline conditions and stimulate antioxidant defense mechanisms. Furthermore, the study reported that application of *P. frigoritolerans* TRM58009 enhanced seed germination, plant growth, chlorophyll content, and antioxidant activities under stress conditions in both pot and hydroponic experiments [128]. Moreover, transgenic petunias expressing the barley-derived iron(III)-phytosiderophore transporter gene *HvYS1* (iron(III)-mugineic acid transporter) displayed increased tolerance to iron deficiency in alkaline soils [127].

Even though these studies highlight the potential of siderophore-producing bacteria to improve plant performance under alkaline stress, the specific contribution of siderophores to these effects remains insufficiently established. Future studies using siderophore-deficient mutants, genetic complementation studies, and purified-siderophore treatments, are needed to establish the causal role of siderophores in improving plant tolerance to alkaline stress.

## 6. Mechanisms of Siderophore-Mediated Stress Tolerance

### 6.1. Siderophore-Mediated Biotic Stress Tolerance

When siderophore-producing bacteria colonize plant roots, they sequester iron from the rhizosphere, restricting the availability of iron to the pathogens. This iron-limiting environment suppresses pathogen growth, thus benefiting plant health indirectly [63]. However, the effectiveness of this mechanism can be affected by the ability of competing microorganisms to acquire iron using their own siderophores or utilize siderophores produced by other species. The selective effects of siderophores depend partly on differences in iron-acquisition systems among microorganisms. Siderophores bind ferric Fe^3+^, but microorganisms vary in their ability to recognize and transport specific siderophore–iron complexes [17,131]. Consequently, a pathogen that cannot efficiently utilize a siderophore produced by a competing microorganism may experience iron limitation, whereas other microorganisms possessing compatible uptake systems may access the bound iron. Some phytopathogens may also exploit siderophores produced by other microorganisms, potentially reducing the effectiveness of iron competition [131]. Therefore, siderophore-mediated interactions do not inherently favor beneficial microorganisms over pathogens; their outcomes depend on siderophore specificity, microbial iron-acquisition capabilities, and environmental conditions.

Beyond this competitive exclusion, siderophore-producing microbes stimulate plant immune responses, triggering induced systemic resistance. In induced systemic resistance, plants experience a “priming” effect, where their immune system is preconditioned to respond faster and more robustly to pathogen attacks. The priming is facilitated by signaling pathways, including the jasmonic acid and ethylene pathways that get activated after exposure to beneficial microbes or their metabolites [132]. Siderophores, acting as microbial metabolites, can initiate this signaling cascade, augmenting the plant’s ability to thwart future infections [133,134]. Furthermore, induced systemic resistance does not involve the direct activation of plant defenses but prepares the plant for enhanced responses when under actual pathogen threat [132].

Pathogen suppression may also involve other microbial antagonistic mechanisms that operate alongside siderophore-mediated iron competition. Siderophores can enhance the synthesis of other antimicrobial metabolites by enhancing the availability of essential minerals to bacteria [79]. A study reported that few bacteria may produce biochemical or volatile compounds detrimental to *R. solani* [65]. A previous study reported that siderophore treatment in plants infected with phytopathogen increased total phenol content and boosted antioxidant enzymes (polyphenol oxidase and peroxidase), which may further defend the plant from various diseases [135]. Therefore, pathogen inhibition by siderophore-producing microorganisms should not automatically be attributed exclusively to iron competition, as other antimicrobial metabolites and plant-mediated defense responses may also contribute to the observed effects.

**Figure 3:**
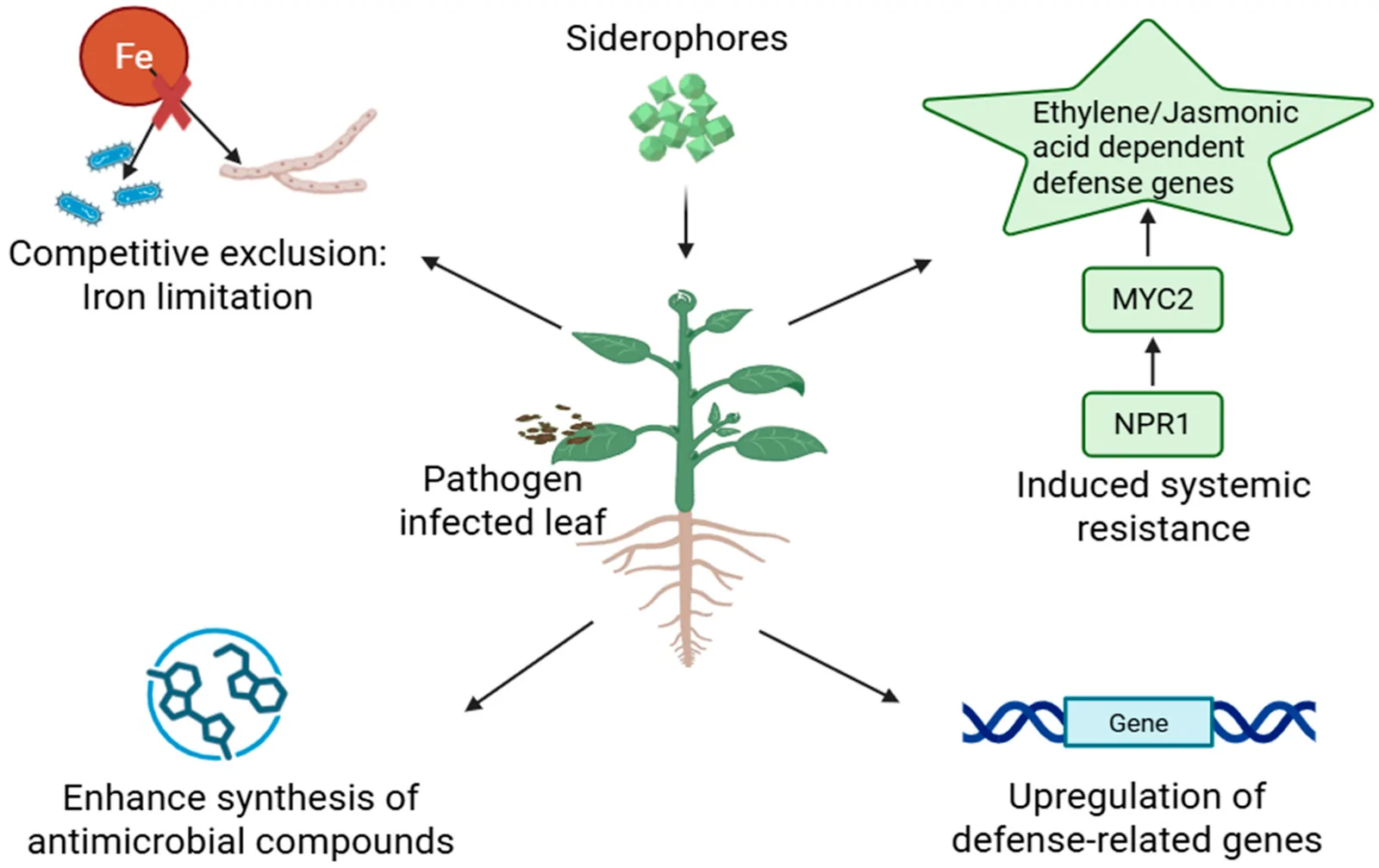
Mechanism of siderophore-mediated control of phytopathogens in plants.

Molecular studies provide further evidence of the plant defense responses associated with siderophore treatment and siderophore-producing microorganisms. The siderophore-producing bacteria can upregulate the expression of genes related to pathogen defense (Figure 3) [65]. A study reported that treating rice leaves affected by *M. oryzae*-mediated blast disease with a hydroxamate-type siderophore from *Pseudomonas putida* BP25 caused defense-related gene upregulation that is associated with salicylic acid (*OsNPR1, OsPDF2.2*, and *OsPR1.1*) and other signaling pathways (*OsCLE*, *OsCSE*, *OsEIN2.4*, *OsGLP3-3*, and *Oshox24*), leading to suppression of the blast disease [135]. The genes responsible for siderophore pyoverdine biosynthesis (*pvdI* and *pvdD*) and chitin-binding protein (gbpA) were also found to be critical for the antifungal activity and biocontrol efficacy of siderophore-producing *P. fluorescens* NBC275 in mitigating the harmful effects of gray mold in pepper fruits [136]. As growth stabilized in *Pseudomonas* sp. P13 exposed to *F. graminearum* T1, the upregulation of hydrogen cyanide and phenazine-1-carboxylate synthesis subsided. Meanwhile, improvement of the TCA cycle led to increased secretion of the siderophores histicorrugatin and pyoverdine, suggesting that this mechanism may have contributed to the *F. graminearum* T1 inhibition [137].

### 6.2. Siderophore-Mediated Heavy Metal Stress Tolerance

The siderophore’s main function is iron chelation, but siderophores also exhibit strong affinities for other metal ions [17]. Siderophores can chelate a wide range of heavy metals and form stable metal complexes, thus reducing their detrimental effects on plants. The secretion of pyoverdine (PVD) by *Pseudomonas aeruginosa* increases in response to higher cadmium concentrations. PVD forms a complex with cadmium that the cell cannot absorb or internalize. This PVD-cadmium complex prevents cadmium(II) from adsorbing onto the cell surface, thereby alleviating cadmium-related oxidative stress, lipid peroxidation, and damage to cell morphology. Hence, the PVD-cadmium complexes improve the cadmium resistance of *P. aeruginosa* [138]. However, because *P. aeruginosa* is an opportunistic human pathogen, its application requires careful biosafety assessment.

Beyond their role in bacterial metal resistance, siderophore-mediated metal interactions can influence metal availability and the effectiveness of phytoremediation. Although siderophores can bind to a plethora of metals, they exhibit high selectivity, facilitating the uptake of specific metals. The selective binding has a crucial role in bacterial metal resistance and contributes to metal bioremediation [8]. When heavy metals bind to free siderophores, they compete with iron ions for chelation, thus reducing the availability of iron. The iron limitation induces the synthesis of additional siderophores, which play a dual role in promoting bacterial or plant growth and mitigating heavy metal toxicity by restricting metal uptake and enhancing stress tolerance mechanisms [139]. Siderophore-producing bacteria improve plant growth in heavy metal-contaminated soils by reducing metal toxicity, improving nutrient availability, and promoting phytoremediation due to increased metal uptake and tolerance [8].

In addition to influencing metal availability and uptake, siderophore-producing bacteria also induce antioxidant responses to limit oxidative stress caused by heavy metals. *Mesorhizobium panacihumi* DCY119T, a siderophore-producing rhizobacterium, has been reported to decrease ROS accumulation and prevent oxidative stress in ginseng seedlings by increasing the expression of ROS scavenging genes and enhancing the production of nonenzymatic antioxidant molecules such as phenols and flavonoids [140].

Furthermore, the application of siderophores can facilitate the formation of phytolith-like structures that help mitigate heavy metal accumulation in plants. In rice, the addition of a hydroxamate siderophore effectively trapped orthosilicic acid and cadmium ions at the third stem node through hydrogen bonding and chelation, with subsequent rapid deposition on the xylem cell wall via hydrophobic interactions. This process immobilized cadmium as phytolith-like CdSiO_3_ particles. Field studies confirmed that this approach promoted cadmium retention in rice stems while reducing cadmium accumulation in rice grains, following hydroxamate siderophore treatment [141].

Siderophore-associated metal tolerance may also extend to aluminium stress, involving both microbial siderophores and plant-derived metal-chelating compounds. *Chaetomium cupreum*, which produces the siderophore oosporein, known for its aluminum detoxification property, has been reported to promote seedling growth and enhance aluminum resistance via two mechanisms: inducing chlorogenic acid production, a phytosiderophore, in *Miscanthus sinensis*, and leveraging the detoxifying capacity of oosporein itself. It indicates that the fungal siderophore may promote phytosiderophore production in plants, thus enhancing their tolerance to metal stress [32].

The roles of microbial siderophores in phytopathogen inhibition and bioremediation are well established; much less is known about their mechanistic contributions to other abiotic stress adaptation. Mainly, the pathways by which siderophores influence ionic homeostasis, osmolyte accumulation, antioxidant activity, and hormonal signaling under alkaline, saline, and drought stress remain poorly understood.

## 7. Factors Affecting Siderophore Production

Microbial siderophore production is governed by an interconnected network of regulatory and environmental factors rather than by individual culture conditions alone. Iron availability is a major determinant of siderophore biosynthesis in many microorganisms, where a low intracellular iron level acts as a key triggering factor for siderophore production [142]. Different transcription factors and regulators such as Fur (ferric uptake regulator), diphtheria toxin regulator (DtxR), and siderophore synthesis orphan regulator (SsoR) participate in the regulation of iron homeostasis and siderophore production in bacteria [143,144]. Different fungi utilize basic leucine zipper (bZIP)-containing regulator (HapX) and GATA repressor (SreA) to control siderophore production [144]. The biosynthesis and regulation of various types of siderophores by microorganisms have been discussed in detail previously [145].

In addition to iron availability, the nutritional and physicochemical environment can influence siderophore production. Carbon and nitrogen sources, carbon-to-nitrogen ratios, pH, temperature, and metal availability have all been associated with changes in siderophore yields under laboratory conditions [6,57,146,147,148,149]. For example, organic acids and other gluconeogenic substrates can support siderophore production in certain microorganisms. The carbon-to-nitrogen ratio is also a key factor affecting siderophore generation. In media containing glucose, higher siderophore yields have been observed at carbon-to-nitrogen ratios of 3:1 and 5:1 [6]. In *Bacillus* sp. PZ-1, maximum hydroxamate-type siderophore production was reported at pH 6.8 with a glucose concentration of 21.84 g/L [147]. Similarly, *Pseudomonas syringae* BAF.1 produced the highest reported yield of a catechol-type siderophore (55.27 μg/mL) when asparagine and glucose were used as nitrogen and carbon sources, respectively, at pH 7 and 30 °C, with a carbon-to-nitrogen ratio of 10:1 [57]. These findings illustrate the importance of nutritional and physicochemical conditions, although the optimal conditions cannot be generalized across microbial species or siderophore classes.

Metal availability can also influence siderophore biosynthesis. In *Brevibacillus brevis* GZDF3, the addition of Fe^3+^ and Cu^2+^ was reported to inhibit siderophore production [146]. Conversely, the presence of particular metal ions has been associated with increased production in some microbial systems; for example, Co^2+^ was reported to enhance pyochelin production in *P. fluorescens* under the conditions investigated [148]. The ZnO nanoparticles have also been associated with increased siderophore production by rhizospheric bacteria *Pseudomonas chlororaphis* O6 [150]. The mechanisms underlying these responses require consideration of the specific organism and experimental conditions.

Other factors are also associated with siderophore production. Chitin addition has been implied to increase siderophore genes in the lettuce rhizosphere microbiome, while phenolic compounds exuded from plant roots under iron-limited conditions can affect the structure of the microbial community to increase the abundance of siderophore-secreting microbes [134,151]. Root exudates may also directly influence microbial metabolism: treatment of *Tsukamurella tyrosinosolvens* with peanut root exudates and compounds including oxalic acid, alanine, and proline increased siderophore production [152]. These findings illustrate that plant-derived compounds can influence siderophore production both through direct effects on microbial physiology and through changes in the composition of the associated microbial community.

These culture-based studies show that pH, carbon and nitrogen sources and availability, and metal ions can influence siderophore production. However, in the rhizosphere, these factors operate within a chemically and biologically complex environment shaped by root exudates, soil properties, and microbial competition. In addition, the production of siderophores should be distinguished from their subsequent activity: siderophore structure, complex stability, metal-binding properties, and iron-release mechanisms can affect whether the produced molecules contribute to iron acquisition or other biological processes. Consequently, optimal conditions identified in culture-based experiments do not necessarily predict siderophore production or activity in agricultural soils.

## 8. Microbial Interactions and Cross-Talk in Siderophore Production and Plant Health

Microbial interactions within the rhizosphere are a complex network of cooperative and competitive relationships that influence siderophore synthesis [153]. These interspecies interactions affect nutrient acquisition, particularly iron availability, thereby shaping microbial community composition and ultimately influencing plant growth and development. Competition for iron among microorganisms can upregulate the expression of genes involved in siderophore biosynthesis and also activate silent biosynthetic gene clusters for producing secondary metabolites like antibiotics [154].

Iron availability is a key determinant of siderophore-mediated microbial competition. When iron is scarce, microorganisms may increase siderophore production to improve iron acquisition, potentially intensifying competition with neighbouring species. A study reported that *Streptomyces coelicolor* exhibited increased production of a unique siderophore, desferrioxamine, when interacting with five other soil actinomycetes, indicating the importance of microbial interplay in increased production and diversification of siderophore production [155]. The interactions can create localized iron scarcity due to competition among microbes. Some microorganisms produce xenosiderophores (siderophores synthesized by neighbouring microorganisms) that differ in their iron-binding properties and accessibility to competing species [131,155]. These xenosiderophores may bind environmental iron and reduce the pool of iron accessible to the competing microorganism. For example, *Amycolatopsis* sp. AA4 produces the siderophore amychelin, which *S. coelicolor* cannot utilize. These “xenosiderophores” chelate environmental iron, thus leaving *S. coelicolor* under iron starvation. The iron limitation acts as a signal to trigger the activation of previously silent biosynthetic gene clusters, leading to an increase in production of siderophores like desferrioxamine and its acylated derivatives [155].

The intensity and outcome of these interactions depend not only on iron availability but also on rhizosphere conditions, including pH, root exudates, and spatial heterogeneity. Rhizosphere pH can influence these interactions by affecting iron solubility, siderophore–iron complex formation, and the activity of siderophore-producing microorganisms [156]. Consequently, the same microbial association may produce different outcomes under different soil pH conditions because both iron availability and microbial physiology can change. Furthermore, root exudates may influence microbial growth, facilitate root colonization, affect plant–microbe interactions, and shape the rhizosphere microbiome [157,158]. Root-derived compounds may therefore indirectly modify siderophore-mediated competition or cooperation by changing which microorganisms are present and how actively they produce siderophores. Spatial heterogeneity is also important because the rhizosphere is not a uniform environment [159]. Root surfaces, root tips, and surrounding soil microsites may differ in exudate composition, oxygen availability, pH, and accessible iron. These localized conditions may create microsites in which siderophore-producing microorganisms gain a competitive advantage, while neighbouring microorganisms may be excluded from access to iron or may exploit siderophores produced by other species when compatible uptake systems are present.

The effects of microbial interactions on siderophore production have been illustrated by studies involving *Staphylococcus aureus*. The *S. aureus* co-culture with *P. aeruginosa* completely inhibited its siderophore production, whereas co-culturing with *E. coli* led to the synthesis of catecholate and carboxylate siderophores. Similarly, when *S. aureus* was co-cultured with *Staphylococcus epidermidis*, it synthesized both the carboxylate and hydroxamate type siderophores [153]. The siderophore-mediated interactions may also help in inhibiting pathogen infections and improving plant growth and development. The in vivo experiments in tomato plants have shown a reduction in disease incidence after inoculation with a consortium of five closely related but non-pathogenic *Ralstonia* strains that produced siderophores inaccessible to the pathogenic *Ralstonia solanacearum*, thereby restricting the pathogen’s growth in the rhizosphere [160]. The hydroxamate siderophore desferrioxamine B (DFO-B) has been observed to affect interspecies interactions by affecting the growth of neighbouring strains of *Streptomyces* spp. For example, *Streptomyces* strain A exhibited phenotypic changes when exposed to DFO-B, leading to transcriptomic changes affecting different metabolic pathways, indicating that siderophores may influence neighbouring microorganisms beyond their role in iron acquisition [161].

Overall, siderophore-mediated microbial interactions are shaped by the combined effects of iron availability, pH, root exudates, and the spatial organization of the rhizosphere. These factors can influence siderophore production, iron competition, microbial community composition, and the potential for pathogen suppression.

## 9. Multi-Omics and Genome Editing Insights into Siderophore-Mediated Plant Stress Tolerance

CRISPR/Cas9-mediated disruption of the *asbA* and *dhbB* genes, associated with petrobactin and bacillibactin biosynthesis, respectively, in *Bacillus mycoides* EC18 revealed differences in plant growth promotion and root colonization among the mutants. The wild-type strain and the *ΔasbA* mutant increased plant biomass and total chlorophyll, whereas the *ΔdhbB* and double-knockout mutants did not exhibit these growth-promoting effects. However, all mutant strains showed reduced root colonization ability compared with the wild-type strain. These findings suggest that the siderophore bacillibactin may contribute to plant growth promotion and affect root colonization. Nevertheless, because the reported findings establish the effects of gene disruption on plant growth and root colonization, rather than directly confirming changes in siderophore production, the specific contributions of petrobactin and bacillibactin require further functional validation [162]. In another study, genome-wide disruption of cis-acting catabolite-responsive elements (cres) in *Bacillus pumilus* altered carbon metabolism and secondary metabolite profiles. KEGG pathway enrichment analysis identified six upregulated genes in the siderophore group nonribosomal peptide biosynthesis pathway (ko01053). In wheat, treatments with the genome-edited strain increased plant height after the seedling stage, shoot fresh weight, and root length during the three-leaf stage. The treatment also reduced *Fusarium graminearum*-associated damage to wheat shoots and roots. Although these findings demonstrate the potential of genome editing to enhance the plant growth-promoting and biocontrol properties of *B. pumilus*, the specific contribution of siderophore biosynthesis to these effects remains unconfirmed and requires functional validation [163]. Similarly, the transcriptomic analysis of the biocontrol strain *Bacillus velezensis* LMY3-5 during co-culture with *Botryosphaeria dothidea*, the major pathogen responsible for kiwifruit soft rot, revealed 114 differentially expressed genes (DEGs), including the upregulation of genes associated with siderophore biosynthesis. The strain exhibited 70.44% biocontrol efficacy against *B. dothidea* in inoculation tests. These findings suggest that siderophores may contribute to the antagonistic activity of *B. velezensis* LMY3-5, but the transcriptomic evidence alone does not establish their direct role in fungal inhibition [164]. Furthermore, RNA-seq analysis revealed a 20-fold downregulation of the *phm* gene cluster, responsible for synthesizing the siderophore phymabactin, in *Paraburkholderia phymatum* under simulated microgravity, resulting in a 66.8% decrease in siderophore production [165]. These findings highlight the sensitivity of siderophore-mediated iron acquisition in this nitrogen-fixing legume symbiont to altered gravitational conditions. Although the implications for plant growth under microgravity require further investigation, maintaining siderophore production in beneficial microorganisms may represent a potential strategy for future space-agriculture systems.

Genome analysis of the halophilic bacterium *Virgibacillus halodenitrificans* ASH15, which promoted sugarcane root and shoot growth and biomass under salt stress, identified the presence of genes encoding siderophore biosynthesis [166]. However, the contribution of these genes to salt-stress tolerance requires functional validation. Furthermore, whole-genome sequencing of the chromium-tolerant *Bacillus tropicus* CRB14 found the presence of siderophore biosynthesis genes, suggesting their potential involvement in plant growth promotion under heavy metal stress, although their functional contribution remains to be established [167]. Genomic analysis of *Bacillus* sp. B25 identified the genetic potential for siderophore production, including bacillibactin and petrobactin biosynthesis, raising the hypothesis that these siderophores may contribute to antagonism against fungal pathogens, which needs further validation through functional studies [168]. Genome analysis of the endophytic bacterium *Serratia* sp. AR11, which enhanced shoot and root growth as well as biomass in pea plants under *Fusarium*-induced stress, exhibited the presence of siderophore biosynthetic genes [169].

Despite their potential, genome-edited microbial products face several challenges before agricultural application. Genetic stability must be established over successive generations, and unintended effects and interactions of the genome-edited microbes with plants or other microorganisms require careful assessment. Off-target effects remain a major challenge in CRISPR-based genome editing, as unintended modifications in non-target genomic regions can reduce editing precision and compromise the safety of engineered microbial strains [170]. In addition, biosafety concerns, which may arise due to unintended genomic outcomes, must be evaluated to identify potential risks to animal and human health, as well as the environment [171]. A clear, consistent regulatory framework is also needed to authorize the use of these genome-edited microbial products across jurisdictions. Furthermore, the genome-edited microbial products may persist, evolve, and interact with diverse biological and environmental systems, complicating risk assessment based solely on static toxicity tests or one-time product characterization [172]. Importantly, the limited availability of field-validation data restricts conclusions about the effectiveness, persistence, and safety of these strains under diverse agricultural conditions. Therefore, long-term stability studies, comprehensive biosafety assessments, and replicated field trials are needed before genome-edited siderophore-producing microorganisms can be considered for practical agricultural use.

## 10. Limitations and Future Perspectives

Despite promising results from laboratory, hydroponic, and greenhouse studies, the agricultural effectiveness of siderophore-based strategies remains insufficiently established under diverse field conditions. Siderophore activity in soil may be influenced by pH, iron availability, competing metal ions, interactions with soil minerals and organic matter, and microbial processes that affect siderophore persistence, uptake, or degradation. Indigenous microorganisms may also consume siderophores or compete with introduced strains, potentially altering their availability and biological effects. These factors, together with differences in soil properties, crop genotypes, climate, and agricultural management, may contribute to variable performance across environments. Accordingly, results obtained under controlled conditions should not be assumed to translate directly into consistent improvements in crop yield or stress tolerance in the field. Robust validation will require replicated field trials across contrasting soil types and growing seasons, accompanied by measurements of plant performance, siderophore persistence, microbial establishment, and relevant soil properties. Commercial translation also requires consideration of formulation stability, shelf life, delivery methods, production costs, regulatory requirements, and compatibility with existing agricultural practices. At present, the purified siderophores cannot be portrayed as established broad-acre agricultural inputs; their practical potential needs to be evaluated separately from that of live microbial inoculants that produce siderophores alongside other beneficial compounds.

Genomic and transcriptomic approaches have substantially advanced the identification of siderophore-biosynthesis genes and the characterization of microbial responses to environmental stress and interactions with plant pathogens. However, changes in the expression of siderophore-associated genes should be interpreted cautiously because differential gene expression provides evidence of association rather than definitive proof of functional requirement. For example, the upregulation of siderophore-biosynthesis genes during microbial co-culture with a plant pathogen may indicate an adaptive response to iron limitation or microbial competition, but it does not independently establish that siderophore production is necessary for pathogen suppression or the associated phenotype. Similarly, the presence of a siderophore-biosynthesis gene cluster in a microbial genome establishes biosynthetic potential but does not necessarily demonstrate its expression, product formation, or contribution to a specific biological outcome. Hence, in future studies the omics findings should follow functional validation experiments. Approaches, including targeted gene deletion, gene overexpression, and complementation of mutant strains, can help establish the contribution of specific genes to siderophore production and associated phenotypes.

Future research should focus on revealing the impact of environmental factors such as pH, temperature, and soil composition on siderophore activity to optimize their possible application in agricultural settings. Additionally, the integration of siderophores with conventional fertilizers requires further studies to increase nutrient use efficiency and promote plant growth. Advances in genetic engineering, such as overexpressing siderophore biosynthetic genes and using CRISPR-Cas9 for targeted genome editing, hold significant potential to increase siderophore production, enhance biocontrol efficacy against plant pathogens, promote plant growth, and increase abiotic stress tolerance. However, many siderophore-producing strains demonstrate promising results under controlled laboratory conditions but face challenges in intricate field environments because of competition with native microbiota. Future research should prioritize strategies to overcome this limitation.

Different types of siderophores, such as hydroxamate, catecholate, and mixed-type siderophores, can differ in iron-binding affinity, which may affect their ability to trigger plant immune signaling. These structural variations may determine the extent of reactive oxygen species generation, hormonal crosstalk, and the activation of downstream defense genes. Understanding the structure–function associations of the siderophores could enable the design or genetic engineering of microbial strains that produce siderophores with better ISR-inducing capacity. Future research should also focus on understanding the crosstalk among the siderophores and plant hormone pathways in regulating plant growth and stress responses.

Future research should prioritize experiments that establish both the mechanistic contribution and field relevance of siderophores. Comparative studies using wild-type strains, siderophore-deficient mutants, complemented strains, and appropriate controls for other plant-growth-promoting traits would help resolve causal relationships. In parallel, replicated field trials across multiple locations and seasons are needed to determine the consistency of effects on crop health, stress tolerance, and yield.

## 11. Siderophores for Sustainable Agriculture

Siderophores hold the potential for the promotion of sustainable agriculture by reducing reliance on chemical fertilizers and pesticides. Siderophores can chelate iron and other micronutrients, such as zinc, copper, and manganese, improving nutrient availability to plants. Moreover, siderophore-producing microorganisms can act as biocontrol agents by inhibiting various phytopathogens through competitive iron sequestration, repellent properties, and by inducing systemic resistance. These activities contribute to the maintenance of soil fertility, microbial biodiversity, and ecological balance. In addition to their nutritional and protective roles, siderophores also play a key role in alleviating abiotic stresses, making them important for climate-resilient agriculture. By incorporating siderophore-producing microorganisms into biofertilizers and biostimulants, crop growth and yield can be improved. Siderophore-producing bacteria may serve as eco-friendly alternatives in biocontrol, thus decreasing the use of chemical pesticides and fertilizers that can contaminate soil and groundwater. Siderophores can help minimize adverse effects on non-target organisms such as earthworms, beneficial insects, and soil microbes by reducing chemical use.

From a policy and commercialization perspective, the integration of siderophore-based fertilizers and stimulants aligns with the United Nations Sustainable Development Goals (SDGs), mainly SDG 2 (Zero Hunger) and SDG 13 (Climate Action). However, widespread application requires addressing various challenges such as strain stability, formulation efficacy, regulatory approval, and field-level consistency. The use of siderophores provides a promising avenue towards environmentally safe agricultural practices. However, challenges associated with the commercialization and large-scale production of siderophore-based products must be addressed before adopting them in agriculture.

## 12. Conclusions

Microbial siderophores have an important role in improving plant tolerance to abiotic and biotic stresses. Siderophores improve plant growth and nutrient uptake, as well as contribute to heavy metal detoxification, phytopathogenic disease inhibition, and other stress tolerance in plants. Their ability to solubilize iron in iron-limited soils, mitigate heavy metal-induced toxicity, and enhance plant defense underscores siderophores’ role in agricultural practice. Furthermore, recent advances in genetic engineering, multi-omics technologies, and microbial inoculant formulations offer new avenues for optimizing siderophore-producing strains for field applications.

Despite their potential, challenges such as environmental variability and limitations in large-scale application must be addressed to harness siderophores’ benefits fully. Future research should focus on improving the stability and efficacy of siderophore-based biofertilizers and biopesticides under diverse agricultural conditions. Additionally, regulatory frameworks need to be developed to support the commercialization of siderophore-based solutions. Combining siderophores with traditional and advanced agronomic practices could increase crop productivity and support sustainable agriculture while reducing dependence on chemical fertilizers and pesticides.

## Data Availability

No new data were created or analyzed in this study. Data sharing is not applicable to this article.
